# Supplementary material for: Mouse Nr2f1 haploinsufficiency unveils new pathological mechanisms of a human optic atrophy syndrome
Source: EMBO Mol Med. 2019 Jul 18;11(8):e10291. doi: 10.15252/emmm.201910291 (PMC6685104; doi:10.15252/emmm.201910291)
Supplement: Supplementary file 1 — Appendix [file EMMM-11-e10291-s001.pdf]

Mouse *Nr2f1* haploinsufficiency unveils new pathological mechanisms of a human optic atrophy syndrome

Bertacchi et al.

APPENDIX FIGURES and LEGENDS

Table of contents:

- **Appendix Figure S1.**

  Nr2f1 reduced levels associated with hyperproliferation and coloboma-like retinal malformations.

- **Appendix Figure S2.**

  Reactive gliosis and reduced myelination in P8 *Nr2f1*-deficient nerves.

- **Appendix Figure S3.**

  Long term effects of Miconazole treatment at post-natal age.

- **Appendix Figure S4.**

  V1 neocortical area shrinkage in *Nr2f1*-deficient brains.

  Appendix Table S1.

  List of P-values.

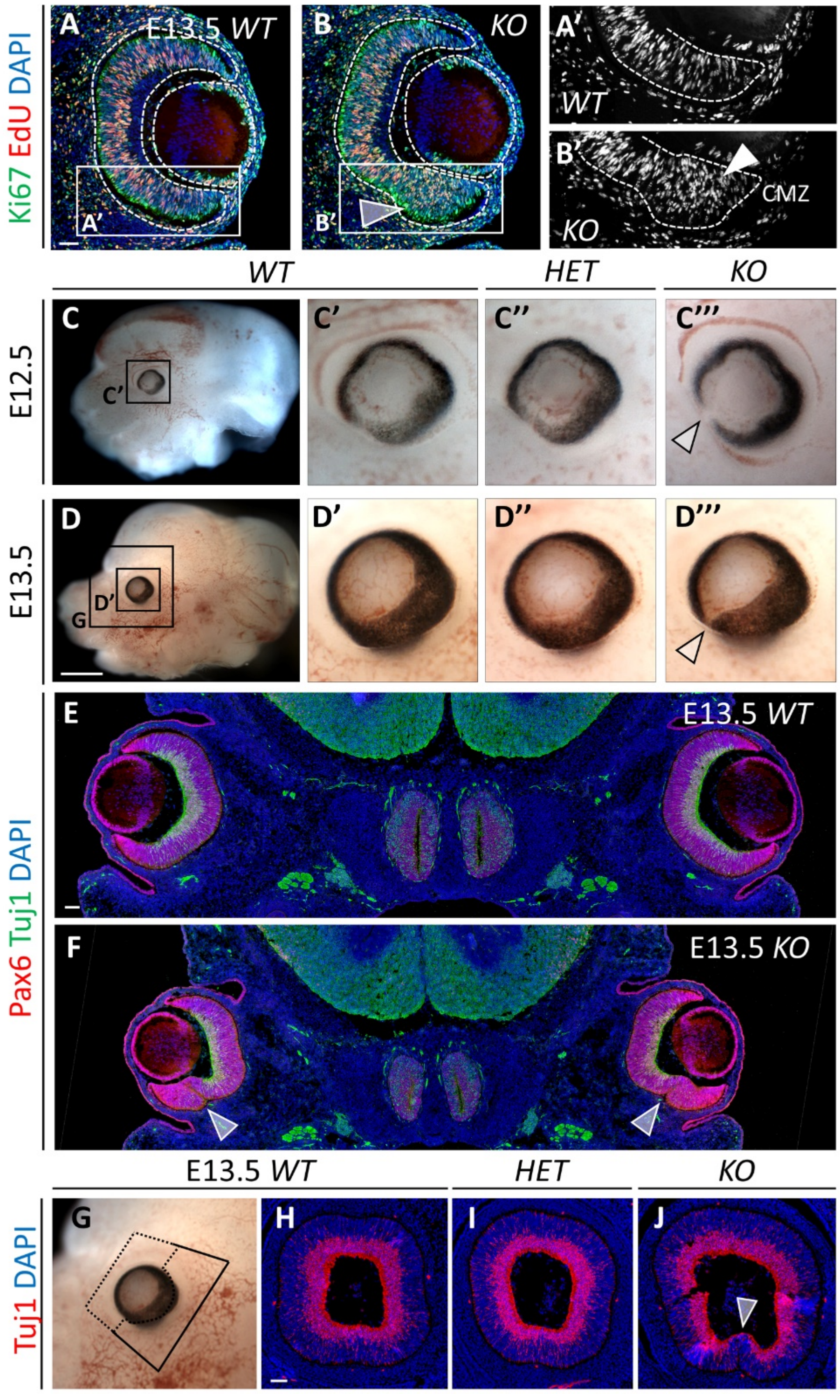

Appendix Figure S1. Nr2f1 reduced levels associated with hyperproliferation and coloboma-like retinal malformations. (**A-B'**) Ki67 (green, proliferative cells) and EdU (red in A,B, grey in A',B'; S-phase proliferating cells) IF on E13.5 *WT* and *KO* optic cup cross-sections showing increased proliferation, particularly in the ventral ciliary marginal zone (CMZ), where Ki67+/EdU+ cells abnormally accumulate in the *KO* (arrowhead in B,B'). (**C-D'''**) Microphotographs of E12.5 (C-C''') and E13.5 (D-D''') *WT*, *HET* or *KO* mouse embryo heads, as indicated. The same embryo head shown in D is magnified in G. Arrowheads in C''' and D''' point to coloboma-like malformations in the ventral *KO* retinas. (**E,F**) Pax6 (NR, red) and Tuj1 (differentiating RG cells, green) IF of E13.5 *WT* (E) and *KO* (F) heads showing expanded CMZ and reduced ventral cell differentiation (arrowheads in F) in *Nr2f1 KO* embryos. (**G-J**) Tuj1 (red) IF of E13.5 eye cross-sections (depicted in G) of *WT* (H), *HET* (I) and *KO* (J) embryos confirming normal ventral fusion in the *KO* retina. Nuclei (blue) were stained with DAPI. Scale bars: 50μm in IFs (A-B',E-J), 1mm in microphotographs (C,D).

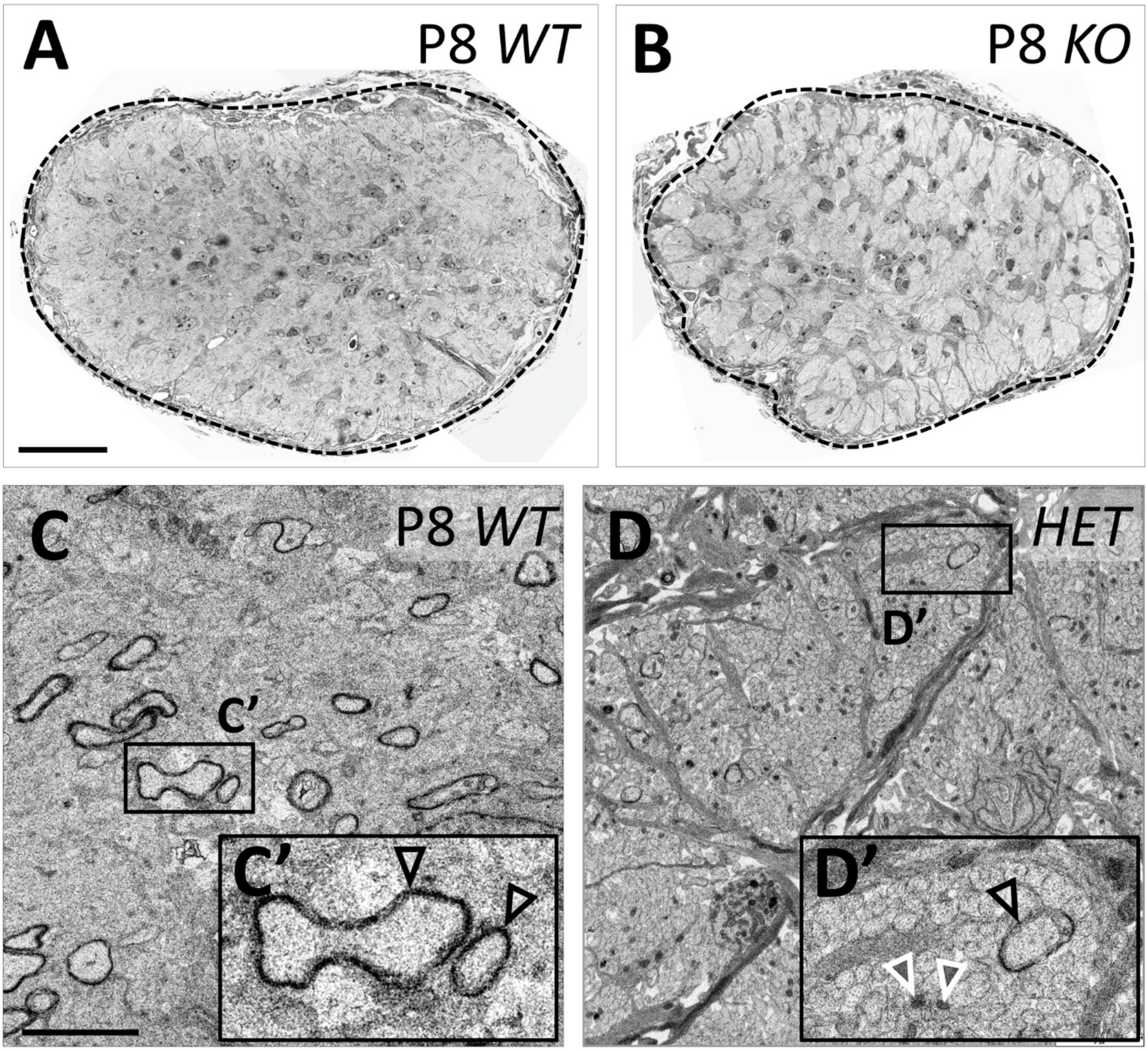

Appendix Figure S2. Reactive gliosis and reduced myelination in P8 *Nr2f1*-deficient nerves. (**A,B**) Electron microscopy (EM) images of P8 *WT* (A) and *KO* (B) mouse ONs displaying an electron-dense cytoplasm in *Nr2f1 KO* nerves. (**C,D**) EM images of P8 *WT* (C) and *HET* (D) mouse ONs showing thick electron-dense astrocytic processes and high number of stress granules (white arrowheads in inset D'). The number of myelinated fibers is lower in *HET* animals (D), than in *WT* littermates (C). Laminae of myelin around axons are often thin and discontinuous in *HET* animals (black arrowheads in C',D'). Scale bars: 100μm in (A,B), 5μm in (C,D).

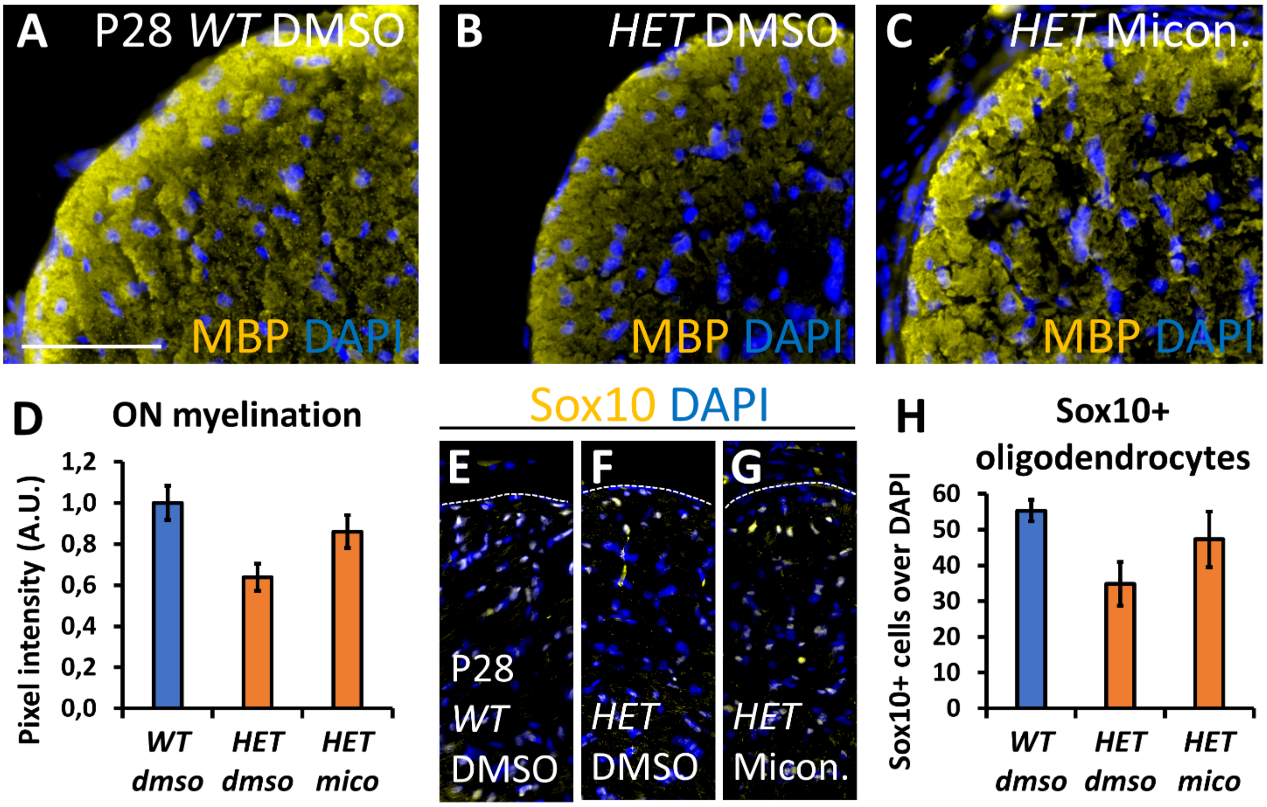

Appendix Figure S3. Long term effects of Miconazole treatment at post-natal age. (**A-D**) MBP (yellow) IF on P28 WT or HET ONs after 6 days of treatment with DMSO (A,B) or Miconazole (C) during the P2-P8 sensitive window. A short treatment with Miconazole partially maintains improved myelination 20 days later (C,D), even if levels failed to reach those of WT (A). EM images of similarly treated samples are shown in Figure 5J-L. (**E-H**) Sox10 (yellow) IF on P28 WT or HET ONs after 6 days of treatment with DMSO (E,F) or Miconazole (G) between P2-P8 showing increased Sox10-expressing cells in treated HET ONs (H). In (D,H), the error bars represent the SEM of the means; N=2. See Supplemental Appendix Table for p-Values. Scale bars: 100µm.

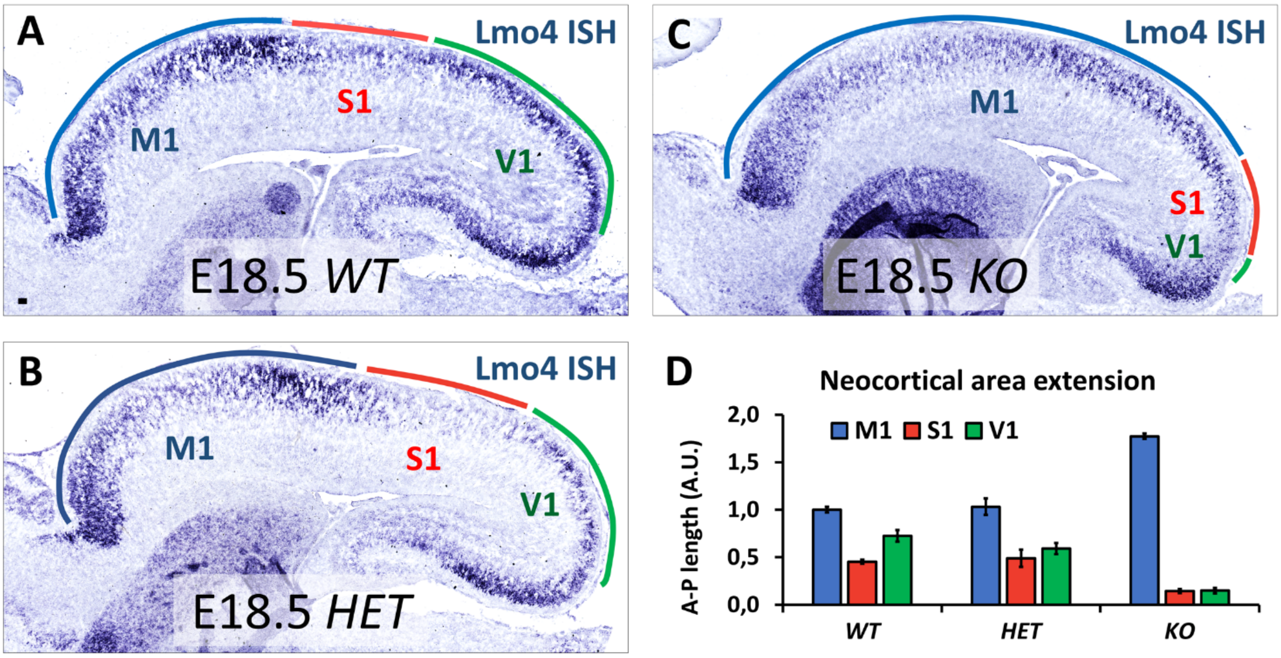

Appendix Figure S4. V1 neocortical area shrinkage in *Nr2f1*-deficient brains. (**A-D**) *In situ* hybridization of *Lmo4 mRNA* in E18.5 *WT* (A), *HET* (B) and *KO* (C) sagittal brain sections. *Lmo4* expression labels the primary motor cortex anteriorly (M1; blue line) and the primary visual cortex posteriorly (V1; green line), leaving the primary somatosensory cortex unlabeled (S1; red line). *Nr2f1* loss shifts neocortical areas, compressing S1 and V1 in the posterior-most region (C); *HET* animals show a slight shift of V1 (B), compared to *WT* (A). Antero-posterior (A-P) length of the different neocortical areas across genotypes is quantified in Graph (D). Data in (D) are represented as mean ± SEM. See Supplemental Appendix Table for p-Values. Scale bars: 100µm.

Appendix Table S1

Figure		sample number	exact p-Value
2	F	n=3-4	E12.5 WT/HET: n.s.=0.1731; E12.5 WT/KO: **=0.0073; E15.5 WT/HET: *=0.01433; E13.5 WT/KO: *=0.02863; E18.5 WT/HET: n.s.=0.141; E18.5 WT/KO: **=0.001395.
2	L	n=3-4	ventral retina WT/HET: *=0.0359; ventral retina WT/KO: *=0.0111; dorsal retina WT/HET and WT/KO: n.s.=0.89 and 0.98, respectively.
2	P	n=3-4	E13.5 WT/HET: n.s.=0.059; E13.5 WT/KO: *=0.01; P7 WT/HET: n.s.=0.465; P7 WT/KO: ***=0.008; P28 WT/HET: ***=0.00011
2	S	n=4-5	P0 WT/HET: n.s.=0.1016; P0 WT/KO: *=0.0421; P8 WT/HET: *=0.015; P8 WT/KO: ***=0.00043; P28 WT/HET: *=0.025.
2	V	n=3-4	E18.5 WT/HET: n.s.=0.0502; E18.5 WT/KO: ***=0,000069; P5 WT/HET: n.s.=0.41; P5 WT/KO: *=0.0396.
3	G	n=3-5	ON retinal side: WT/HET: *=0.0339; WT/KO: *=0.0108; ON chiasmal side: WT/HET: n.s.=0.2264; WT/KO: *=0.0242.
3	H	n=3-5	ON retinal side: WT/HET: n.s.=0.1985; WT/KO: *=0.0462; ON middle region: WT/HET: *=0.0237; WT/KO: **=0.0022; ON chiasmal side: WT/HET: *=0.0171; WT/KO: **=0.0031.
3	I	n=3-5	ON retinal side: WT/HET: **=0.0015; WT/KO: **=0.0039; ON middle region: WT/HET: *=0.026; WT/KO: **=0.0053; ON chiasmal side: WT/HET: n.s.=0.364; WT/KO: *=0.0381.
3	L	n=3-5	P7 WT/HET: *=0.02797; P7 WT/KO: ***=2.336E-06.
3	T	n=3	P28 WTdmso/HETdmso: *=0.0134
4	C	n=4-5	p-Value not tested
4	J	n=4-5	chiasmal region ("c"): WT/HET: *=0.026; WT/KO: **=0.0033; optic nerve ("ON"): WT/HET: ***=3.17E-09; WT/KO: ***=2.88E-09
4	N	n=3	P8 WT/HET: ***=0.00033; P8 WT/KO: ***=2.5E-09; P8 HET/KO: ***=1.8E-05.
4	Q	n=3	P28 WT/HET: ***=0.000519
5	B	n=3	HETdmso/HETmiconazole: **=0.00134
5	C	n=3	WTdmso/HETdmso: *=0.01; WTdmso/KOdmso: ***=0.00169; WTdmso/HETmiconazole: n.s.=0.0975.
5	H	n=2	WTdmso/HETdmso: n.s.=0.053; WTdmso/HETmico: ***=0.0037; HETdmso/HETmico: ***=0.000024.
5	I	n=2	WTdmso/HETdmso: n.s.=0.4; WTdmso/HETmico: ***=0.00025; HETdmso/HETmico: ***=0.000025.
5	M	n=2	g-Ratio: WTdmso/HETdmso: ***=0.00029; WTdmso/HETmico: n.s.=0.055; HETdmso/HETmico: *=0.049.
5	Q	n=2	p-Value not tested
6	D	WT=13; HET=18	exact p-Value in Main text and/or Material and Methods; LGN latency WT/HET: ***=0.001; SC latency WT/HET: *=0.046; V1 latency WT/HET: **=0.005.
6	F	WT=13; HET=18	exact p-Value in Main text and/or Material and Methods; WT/HET: n.s.=0.703
6	G	WT=13; HET=18	exact p-Value in Main text and/or Material and Methods; WT/HET: n.s.=0.473
6	H	WT=13; HET=18	exact p-Value in Main text and/or Material and Methods; WT/HET at 6th conditioning session: *=0.026
6	I	WT=13; HET=18	exact p-Value in Main text and/or Material and Methods; WT/HET: ***=0.001.
EV1	B	n=3	p-Value not tested
EV1	C	n=3	p-Value not tested
EV2	D	n=4-5	p-Value not tested
EV2	E	n=4-5	p-Value not tested
EV6	B	n=3-4	WT/KO: n.s.=0.16.
EV6	D	n=3-4	WT/KO: n.s.=0.56.
EV6	H	n=3-4	p-Value not tested
EV6	I	n=3-4	p-Value not shown in graph; WT/HET: *=0.01396; WT/KO: ***=0.00075; HET/KO: n.s.=0.21.
EV7	D	n=2	p-Value not shown in graph; WTdmso/HETdmso: **=0.00216; WTdmso/HETmico: n.s.=0.24; HETdmso/HETmico: *=0.045.
EV7	H	n=2	p-Value not shown in graph; WTdmso/HETdmso: *=0.03306; WTdmso/HETmico: n.s.=0.5069; HETdmso/HETmico: n.s.=0.2212.
EV8	C	n=3-4	P0 WT/HET: n.s.=0.11; P0 WT/KO: **=0.0032
EV8	F	n=3-4	P7 WT/HET: *=0.019; P7 WT/KO: ***=1,43E-07
EV8	I	WT/HET=2; KO=3	ipsi WT/HET: *=0.018; ipsi WT/KO: *=0.0165; contra WT/HET: n.s.=0.17; contra WT/KO: ***=0.000022.
EV9	D	n=2	p-Value not shown in graph; M1, S1 and V1 WT/HET: n.s.=0.68; 0.60; 0.14 respectively; M1, S1 and V1 WT/KO: ***=1.33E-14; 3.64E-10; 3.74E-08 respectively.
