# Supplementary material for: Mouse Nr2f1 haploinsufficiency unveils new pathological mechanisms of a human optic atrophy syndrome
Source: EMBO Mol Med. 2019 Jul 18;11(8):e10291. doi: 10.15252/emmm.201910291 (PMC6685104; doi:10.15252/emmm.201910291)
Supplement: Supplementary file 2 — Expanded View Figures PDF [file EMMM-11-e10291-s002.pdf]

Expanded View Figures

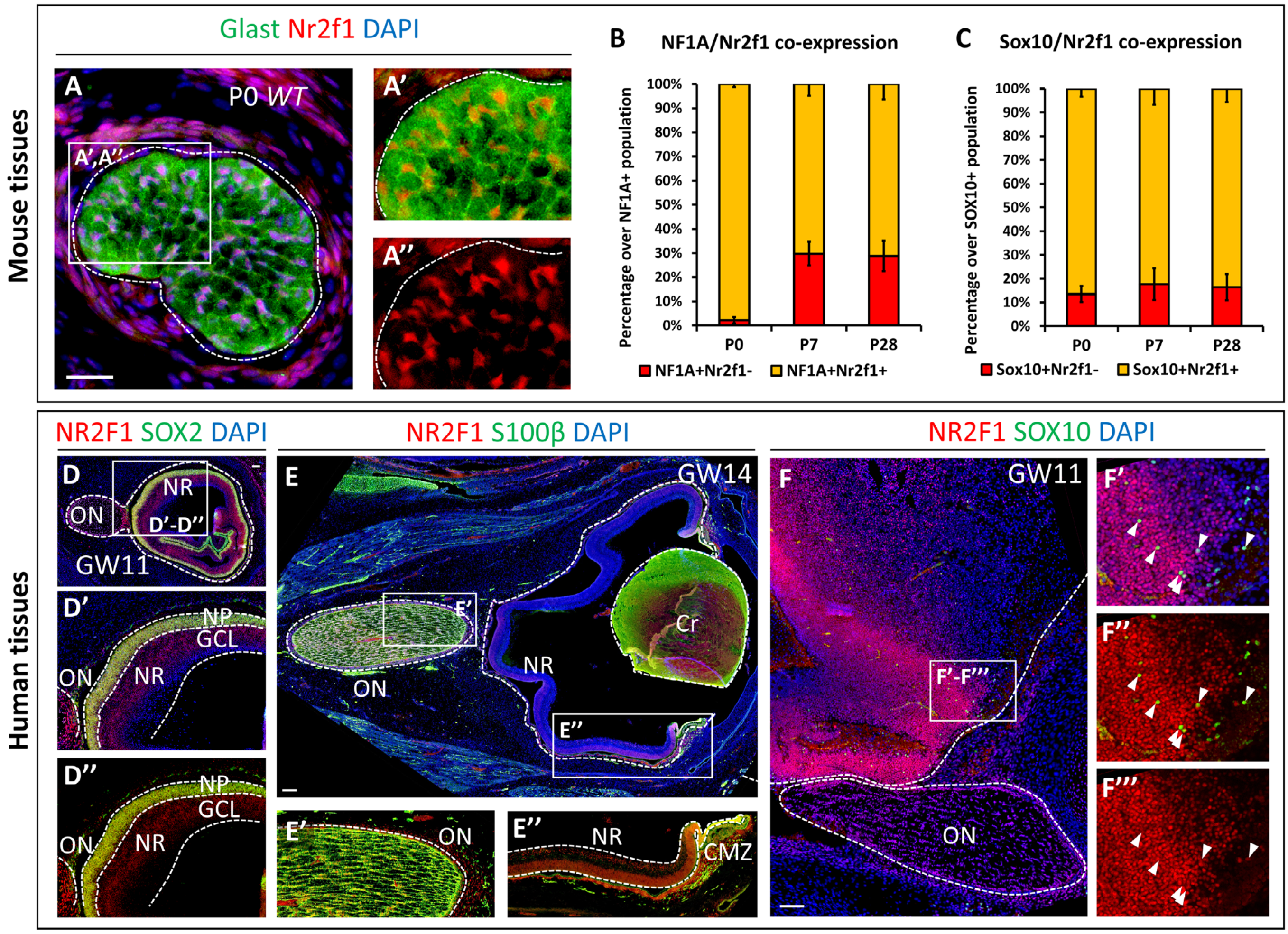

Figure EV1. Nr2f1/NR2F1 expression in mouse and human retina and optic nerve.

A–A" Nr2f1 (red) and Glast (green, astrocyte precursors) immunofluorescences (IF) in a wild-type (*WT*) P0 mouse optic nerve (ON) showing that almost all Glast$^+$ astrocytes are co-labeled with Nr2f1 (insets A', A").

B Graph illustrating the percentage of double NF1A$^+$/Nr2f1$^+$ astrocytes (yellow) over the total NF1A$^+$ population in P0, P7 and P28 mouse ONs.

C Graph showing the percentage of double Sox10$^+$/Nr2f1$^+$ oligodendrocytes (yellow) over the total Sox10$^+$ population, in P0, P7 and P28 mouse ONs.

D–D" NR2F1 (red) and SOX2 (green, NR progenitors) IF of gestational week (GW) 11 human eye, showing high NR2F1 expression in all NR progenitors. GCL, ganglion cell layer; NP, neural progenitor layer; NR, neural retina.

E–E" NR2F1 (red) and S100β (green) IF in a sagittal GW14 eye section showing NR2F1 expression in almost all astrocytes of the ON (E'), in the NR and ciliary marginal zone (CMZ; E"). High-magnification views of the ON are shown in Fig 1N–N". Cr, lens crystal.

F–F"' NR2F1 (red) and SOX10 (green, oligodendrocyte precursors) IF in a sagittal GW11 human brain section (proximal ON and basal forebrain region) illustrating the presence of SOX10$^+$ oligodendrocyte progenitors at the ventricular zone and in proximity of the ON (arrows in F'–F"').

Data information: Nuclei (blue) were stained with DAPI. In (B, C) data were normalized to the total NF1A$^+$ or Sox10$^+$ cells and represented as mean ± SEM; $N = 3$. Scale bars: 50 μm in (A), 100 μm in (D–F).

© 2019 The Authors

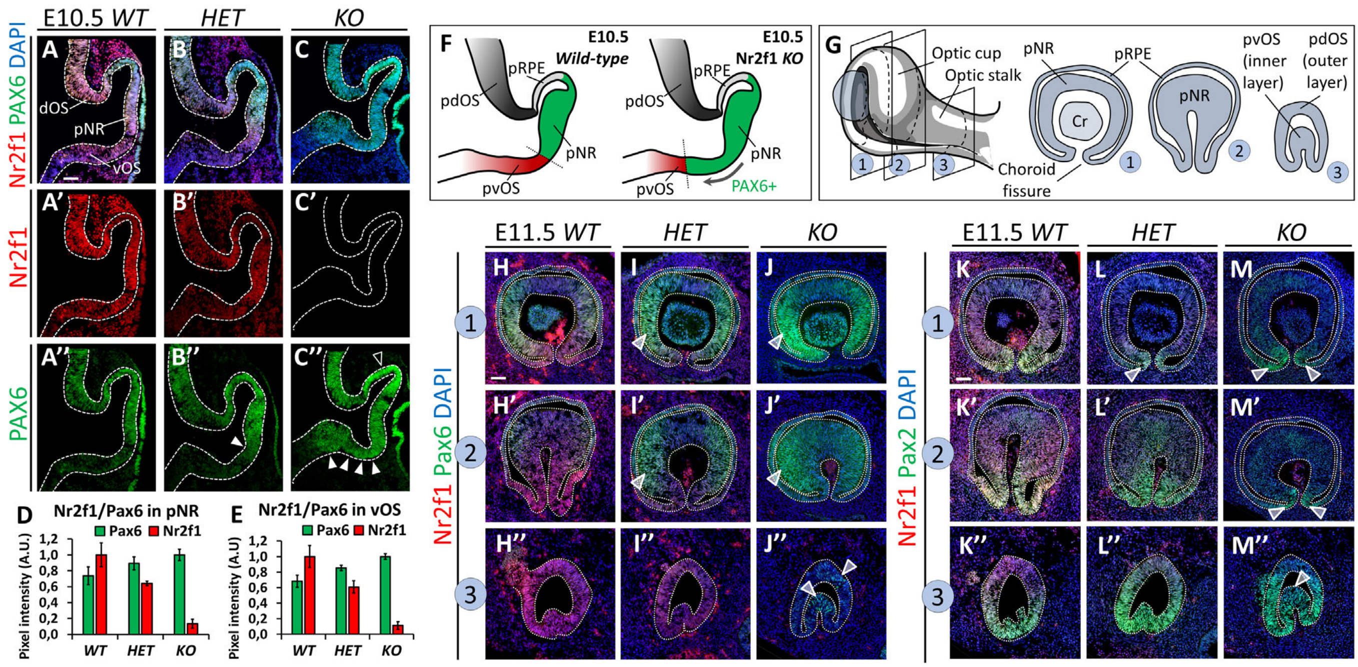

Figure EV2. Nr2f1-dependent control of early OS/NR molecular domains.

A–C" Nr2f1 (red) and Pax6 (green, NR) IF on sagittal sections of E10.5 optic vesicles in wild-type (*WT*), heterozygous (*HET*), and full knock-out (*KO*) embryos. (A'–C') Note the progressive reduction of Nr2f1 protein levels from *WT* to *KO*. (A"–C") Full and empty arrowheads point to increased Pax6 expression in the ventral and dorsal regions, respectively, of the optic vesicle.

D, E Pixel intensity quantification of Pax6 and Nr2f1 IF in presumptive neural retina (pNR; D) and ventral optic stalk (vOS; E) of E10.5 embryos confirming an inverse relationship between Nr2f1 and Pax6 protein levels. Data represent the mean ± SEM; N = 4–5.

F Scheme illustrating the expansion of Pax6^{+} pNR domain (green) toward the pvOS domain (red) in *Nr2f1 KO* mutants. pdOS, presumptive dorsal optic stalk; pRPE, presumptive retinal pigmented epithelium.

G Scheme depicting an E11.5 mouse eye primordium and the structures visible after cross-section at different levels, from distal (1) to middle (2) to proximal (3).

H–J" Nr2f1 (red) and Pax6 (green, NR) IF on E11.5 *WT*, *HET*, and *KO* optic cups, at the levels indicated above showing the inverse correlation between Nr2f1 and Pax6 expression levels from *WT* to *KO* (arrowheads in I, J, I', J'). Note the presence of Pax6^{+} cells in the proximal region of the pvOS of *KO* animals (J"), suggesting that pNR cells substitute pvOS cells along the OS axis.

K–M" Nr2f1 (red) and Pax2 (green) IF on E11.5 *WT, HET,* and *KO* optic cups at the levels indicated above and showing reduced Pax2 expression and reciprocal Pax6 is upregulation in the ventral region of the pNR of *HET* and *KO* animals (arrowheads in L, M, M').

Data information: Nuclei (blue) were stained with DAPI. Scale bars: 50 μm.

 © 2019 The Authors

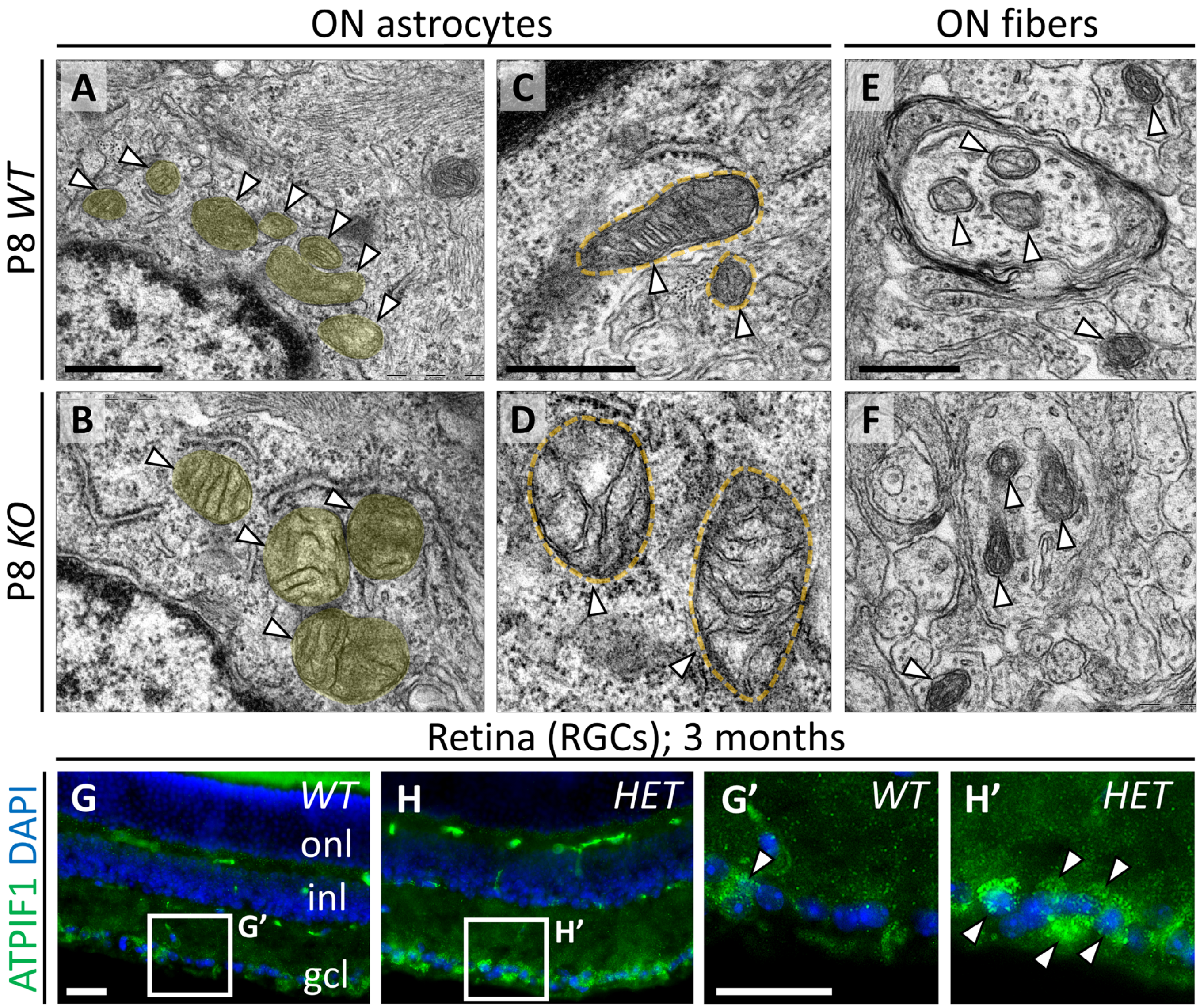

Figure EV3. Mitochondrial hypertrophy in ON astroglia and RGC somata.

A–F Electron microscopy (EM) images of P8 *WT* (A, C, E) and *KO* (B, D, F) mouse ONs displaying the structure of mitochondria in both astrocyte cytoplasm (A–D) and RGC axons (E, F). The average size of glia mitochondria (white arrowheads in A–D; highlighted in yellow in A, B) is increased in *KO* ONs (1.78 ± 0.15 times bigger than *WT*; $P = 0.0018$), additionally showing abnormal *cristae* (D). On the contrary, their structure/size seems normal in axons (E, F).

G–H' ATPIF1 (pan-mitochondria OxPhos marker, green) IF in the retina of 3-month-old *WT* (G, G') and *HET* (H, H') animals, showing intense staining in *HET* ganglion cell layer (gcl). High-magnification images show increased mitochondrial staining in *HET* GCL cytoplasm (arrows in H').

Data information: Nuclei (blue) were stained with DAPI. Scale bars: 500 nm in (A–F), 50 µm in (G–H'). onl, outer nuclear layer; inl, inner nuclear layer.

© 2019 The Authors

Figure EV4. Oligodendrocyte maturation is defective in *Nr2f1*-deficient nerves.

A, B Sox10 (oligodendrocyte marker, red) and Ki67 (proliferation marker, green) IF in P7 *WT* and *KO* ONs showing normal proliferation of single $Sox10^+$ (non-proliferative, red) and double $Sox10^+$/$Ki67^+$ (proliferative, yellow) oligodendrocytes, as quantified in (B).

C, D Sox10 (oligodendrocyte marker, red) and cleaved Caspase3 (apoptotic marker, green) IF in P7 *WT* and *KO* ON showing an equivalent number of double $Sox10^+$/$Caspase3^+$ apoptotic oligodendrocytes (arrowheads) in both genotypes, as quantified in (D).

E–I Sox10 (oligodendrocyte marker, green) and MBP (fully differentiated oligodendrocyte marker, red) IF in P7 *WT* (E), *HET* (F), and *KO* (G) ONs illustrating strong reduction of oligodendrocytes on the retinal side of *HET* and *KO* ON. Percentages of $Sox10^+$ cells and their distribution along the ON are quantified in (H). (I) Ratio of MBP^+/MBP^- oligodendrocytes.

J Schematic summary of Nr2f1 role in the $Sox10^+$ oligodendrocyte population. Nr2f1 controls the development of oligodendrocyte precursors, their migration along the ON and their terminal differentiation.

Data information: Nuclei (blue) were stained with DAPI. In (B, D, H, I), data are represented as mean ± SEM; N = 3–4. Scale bars: 50 μm.

 © 2019 The Authors

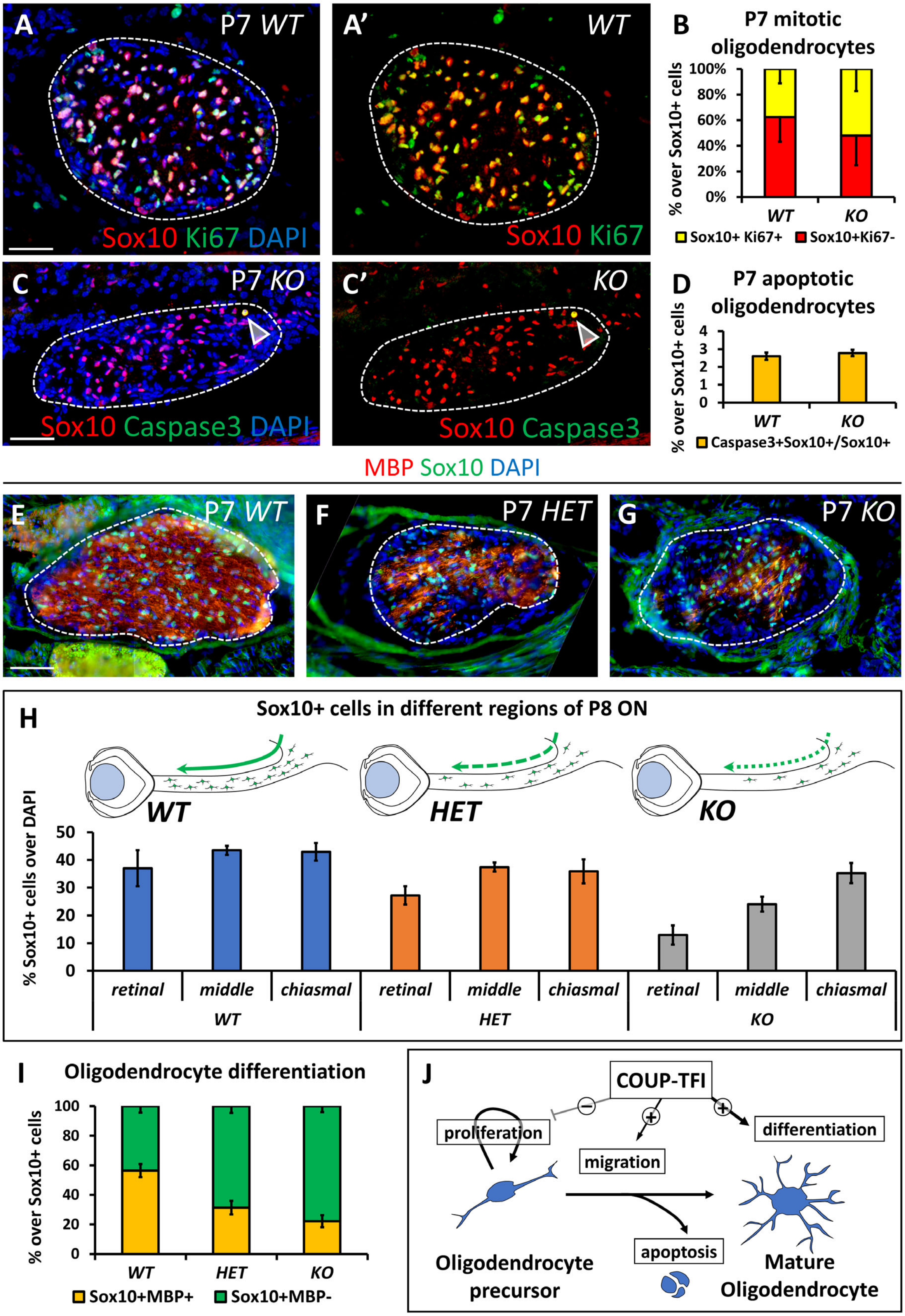
A P7 WT
Sox10 Ki67 DAPI
A' WT
Sox10 Ki67
B P7 mitotic oligodendrocytes
% over Sox10+ cells
100%
80%
60%
40%
20%
0%
WT
KO
Sox10+ Ki67+
Sox10+Ki67-
C P7 KO
Sox10 Caspase3 DAPI
C' KO
Sox10 Caspase3
D P7 apoptotic oligodendrocytes
% over Sox10+ cells
4
3
2
1
0
WT
KO
Caspase3+Sox10+/Sox10+
MBP Sox10 DAPI
E P7 WT
F P7 HET
G P7 KO
H Sox10+ cells in different regions of P8 ON
WT
HET
KO
% Sox10+ cells over DAPI
50
40
30
20
10
0
retinal
middle
chiasmal
retinal
middle
chiasmal
retinal
middle
chiasmal
WT
HET
KO
I Oligodendrocyte differentiation
% over Sox10+ cells
100
80
60
40
20
0
WT
HET
KO
Sox10+MBP+
Sox10+MBP-
J COUP-TFI
proliferation
migration
differentiation
apoptosis
Oligodendrocyte precursor
Mature Oligodendrocyte

Figure EV4.

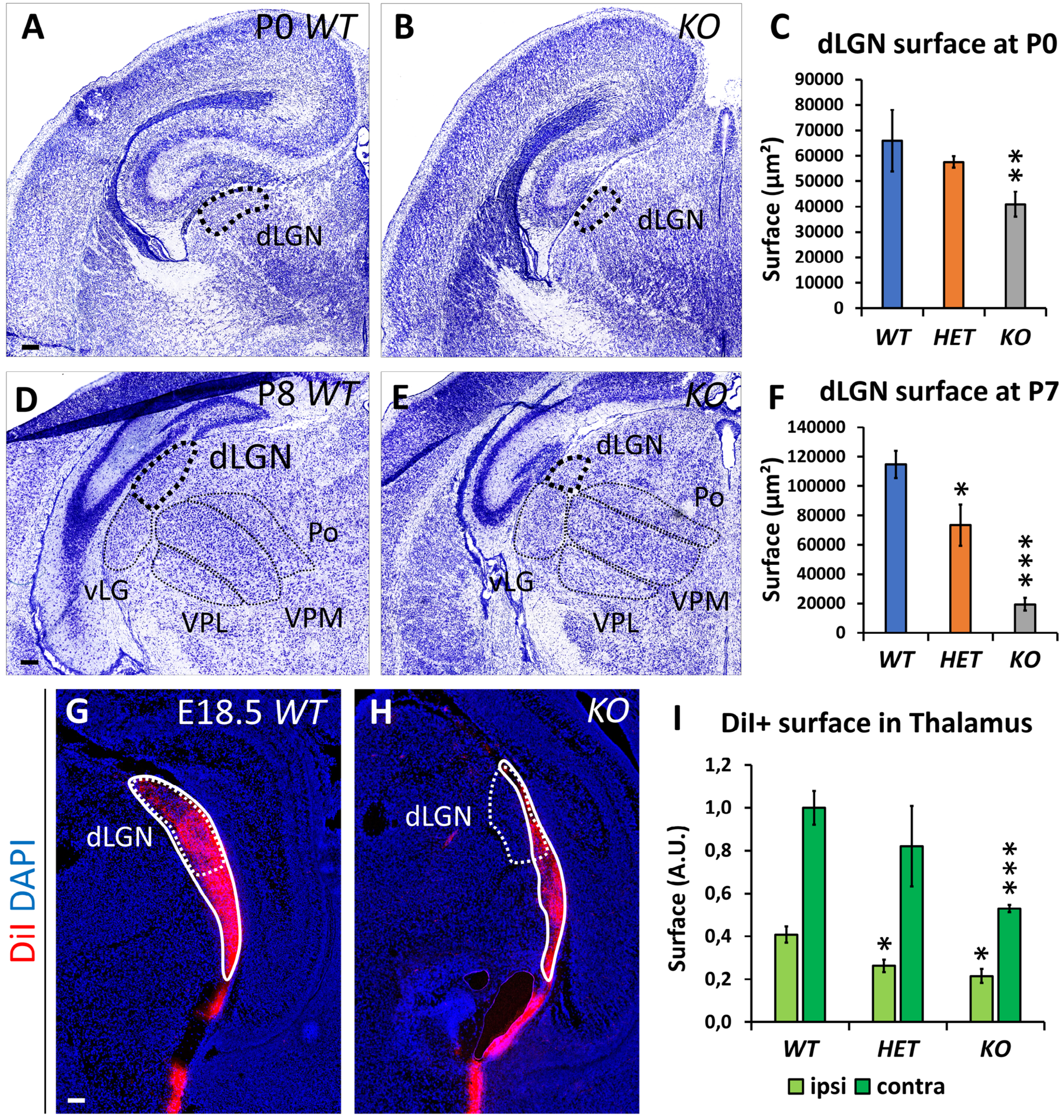

Figure EV5.

 © 2019 The Authors

Figure EV5. Visual system impairment in *Nr2f1* mutants at thalamic level.

A–C Nissl staining of P0 *WT* (A) and *KO* (B) brain coronal sections, showing strong reduction of the dorsolateral geniculate nucleus (dLGN; dotted black line) in the *Nr2f1 KO* thalamus, compared to *WT*. Area quantification of *WT*, *HET,* and *KO* dLGN in P0 brains is shown in (C).

D–F Nissl staining of P8 *WT* (D) and *KO* (E) brain coronal sections confirming persistent and exacerbated reduction of the dLGN (thicker dotted line) in *KO* brains, as quantified in (F). vLGN, ventrolateral geniculate nucleus; Po, posterior nucleus; VPL, ventral posterolateral nucleus; VPM, ventral posteromedial nucleus.

G, H Fluorescent axonal labeling (red staining, highlighted by white line) of optic tract fibers after injection of DiI crystals in the eye showing reduced innervation of *KO* fibers in the dLGN. White dotted lines highlight the size of the dLGN.

I Quantification of the ipsi- (light green) and contra-lateral (dark green columns) fibers reaching the dLGN.

Data information: Nuclei (blue) were stained with DAPI. In (C, F, I), data are represented as mean ± SEM. Statistical significance was obtained by ANOVA (*$P < 0.05$; **$P < 0.01$; ***$P < 0.001$). N = 3–4. Scale bars: 100 μm.

© 2019 The Authors
